# Association of decreased serum sTREM-1 level with the severity of coronary artery disease: Inhibitory effect of sTREM-1 on TNF-α- and oxLDL-induced inflammatory reactions in endothelial cells

**DOI:** 10.1097/MD.0000000000004693

**Published:** 2016-09-16

**Authors:** Daopeng Dai, Weixin Xiong, Qin Fan, Haibo Wang, Qiujing Chen, Weifeng Shen, Ruiyan Zhang, Fenghua Ding, Lin Lu, Rong Tao

**Affiliations:** a Department of Cardiology, Rui Jin Hospital; b Institute of Cardiovascular Diseases, Shanghai Jiaotong University School of Medicine, Shanghai, People's Republic of China.

**Keywords:** CAD, diabetes mellitus, human umbilical vein endothelial cells, inflammatory reaction, soluble TREM-1

## Abstract

Soluble triggering receptor expressed on myeloid cells 1 (sTREM-1) is closely involved in autoimmune diseases and inflammatory reactions. We aimed to investigate whether serum sTREM-1 is related to coronary artery disease (CAD) and to evaluate the biological effects of sTREM-1 in cell experiments.

This cross-sectional study included 263 consecutive patients with angiographically documented CAD, who were admitted for diagnosis and interventional treatment of CAD (CAD group), with 162 participants without CAD serving as controls (control group). Serum levels of sTREM-1 and high sensitivity C reactive protein (hsCRP) were determined in all participants. In cell experiments, the influence of sTREM-1 on tumor necrosis factor-α (TNF-α)- or oxidized low-density lipoprotein (oxLDL)-induced inflammatory reactions was evaluated in human umbilical vein endothelial cells (HUVECs).

Serum level of sTREM-1 was significantly lower in CAD patients than in controls (*P* < 0.001). sTREM-1 values were related to the number of diseased coronary arteries (Spearman *r* = −0.413, *P* < 0.001) and the severity represented by Gensini score (Pearson *r* = −0.336, *P* < 0.001). Multivariable logistic regression analysis revealed that decreased sTREM-1 were independent determinants of CAD (OR = 0.428, *P* < 0.001). In cell experiments, recombinant sTREM-1 protein concentration-dependently inhibited the expression of IL-1β, IL-6, TNF-α, VCAM-1, and ICAM-1 induced by TNF-α or oxLDL in HUVECs.

This study demonstrates that decreased serum sTREM-1 level is significantly associated with the presence and severity of CAD. sTREM-1 restrains inflammatory reaction in endothelial cells, suggesting that it might be a potential vascular protective factor.

## Abstract 2

Supplemental Digital Content is available in the text

## 1 Introduction

Atherosclerosis is a chronic vascular inflammatory disease.^[1,2]^ In atherogenic process, monocytes/macrophages are activated, and increased cytokine and chemokine production from inflammatory cells and vascular wall are solicited by cardiovascular risk elements to participate in disease pathophysiology.^[3,4]^ The triggering receptor expressed on myeloid cells 1 (TREM-1) is a member of the immunoglobulin superfamily identified on monocytes, neutrophils, macrophages, and endothelial.^[5–7]^ This protein contains an Ig-like extracellular domain and a short cytoplasmic tail without signaling motif.^[5]^ TREM-1 activates cells through DNAX-activating protein of 12 kDa, an associated signal transduction molecule, and promotes secretion of interleukine-1β (IL-1β), tumor necrosis factor-α (TNF-α), and monocyte chemotactic protein-1. Moreover, TREM-1 facilitates the production of inflammatory cytokines through induction of Toll-like receptor and NOD-like receptor pathways.^[8–10]^ Blockage of TREM-1 protects against LPS-induced shock in mice and reduces immune-associated diseases in TREM-1^−/−^ mice.^[11–13]^ These findings suggest that TREM-1 is closely involved in inflammatory reaction and relevant pathophysiology.

TREM-1 is also produced in a soluble form, which lacks the transmembrane domain.^[14,15]^ Soluble triggering receptor expressed on myeloid cells 1 (sTREM-1) proteins are detected in patients suffering from severe infectious diseases including pneumonia, sepsis, and chronic bowel disease.^[16,17]^ Elevated level of sTREM-1 is associated with the disease severity and adverse outcome in patients with sepsis and lower respiratory tract infection.^[18,19]^

Of note, serum sTREM-1 level is significantly associated with myocardial injury in patients with severe sepsis.^[20]^ Moreover, sTREM-1 is obviously elevated in patients with acute myocardial infarction, and its value is an independent predictor of death following myocardial infarction.^[21]^ Nevertheless, the relation between sTREM-1 and coronary artery disease (CAD) remains unclear. Golovkin et al^[22]^ found certain TREM-1 gene polymorphisms were significantly associated with CAD in Russian population, indicating a potential role of TREM-1 in the progress of CAD. Based upon above-mentioned information, we hypothesized a potential association of sTREM-1 with CAD, and hence analyzed serum sTREM-1 level in controls and patients with CAD to test this hypothesis. To probe the biological effects of sTREM-1, we treated endothelial cell human umbilical vein endothelial cells (HUVECs) by TNF-α and oxidized low-density lipoprotein (oxLDL) with or without addition of sTREM-1 to assess the influence of this protein on inflammatory reactions.

## 2 Methods

The study protocol was approved by the Ethics Committee of Rui Jin Hospital, Shanghai Jiaotong University School of Medicine, and written informed consent was obtained from all participants.

### 2.1 Study population

This study included 263 consecutive patients with coronary angiographically diagnosed as CAD from September 2014 to March 2015. To avoid confounding data, patients with acute myocardial infarction, congenital heart disease, cardiomyopathy, valvular heart disease, acute and chronic viral or bacterial infection, tumors, rheumatoid arthritis, and other connective tissue diseases were excluded. Patients with type 1 diabetes mellitus were also excluded based on C-peptide measurement. Type 2 diabetes mellitus was identified as 2 fasting plasma glucose levels ≥7.0 mmol/L or 2 2-hour postprandial plasma glucose readings ≥11.1 mmol/L after a glucose load of 75 g or 2 casual glucose readings ≥11.1 mmol/L, or taking oral hypoglycemic drugs or parenteral insulin.^[23]^ CAD patients were documented based on the angiographic examination (luminal diameter narrowing ≥50%).^[24]^ Patients with significant CAD were further classified according to the number of diseased coronary arteries (1-, 2-, or 3-vessel disease).

A total of 162 subjects with no evidence of cardiovascular diseases served as controls, who received annual physical check-up in our hospital. For each of them, we took a detailed medical and family history, and collected fasting blood samples during an outpatient visit. All subjects had normal chest X-ray examination, carotid artery ultrasound examination, resting electrocardiogram, and an exercise stress test. Upon interrogation and data collection, all had no history of any cardiovascular diseases, including any past history of angina/myocardial infarction.

### 2.2 Coronary angiography and Gensini score

Selective coronary angiography was performed through femoral or radial artery approach by interventional cardiologists blinded to the study protocol. Significant CAD was diagnosed visually if luminal diameter narrowing ≥50% was present at a major epicardial coronary artery, and left main coronary artery narrowing ≥50% was considered as 2-vessel disease.^[24]^ Gensini score was calculated according to the method presented by Gensini.^[25]^

### 2.3 Biochemical investigation

Blood samples were collected after an overnight fasting from all patients. After coagulation (30 minutes at room temperature) and being centrifuged at 3000 rpm for 15 minutes, blood serum was then collected and stored at −80 °C for analysis. Serum glucose, creatinine, blood urea nitrogen, uric acid, total cholesterol, low-density lipoprotein-cholesterol, high-density lipoprotein cholesterol, lipoprotein (a), apoprotein A, apoprotein B, and triglycerides were measured with standard laboratory techniques. sTREM-1 level was determined with a commercially available ELISA kit (Human TREM-1 duoset, DY1278B, R&D System, Minneapolis, MN) according to its protocol. The detection limit for sTREM-1 was 93.8 to 6000 pg/mL with an interassay coefficient of variation <10%.

### 2.4 Antibodies and reagents

TNF-α (ab66579) and IL-6 (ab6672) primary antibodies were purchased from Abcam (Cambridge, UK). IL-1β (sc-7884) was obtained from Santa Cruz Biotechnology (Dallas, TX). VCAM-1 (12367s) and ICAM-1 (4915s) antibodies were purchased from Cell Signaling Technology (Beverly, MA). HRP-conjugated antibody (Cell Signaling Technology, Beverly, MA) was used as secondary antibody. Recombinant human sTREM-1 (1278-TR-050) and recombinant human TNF-α (210-TA-050) were obtained from R&D system (Minneapolis, MN). OxLDL was purchased from abD serotec (Oxford, UK).

### 2.5 Cell culture

The HUVECs (395z050, PromoCell, Germany) were cultured in a humidified atmosphere consisting of 5% CO_2_ and 95% air at 37 °C in Endothelial Cell Growth Media 2 (C-22011, PromoCell, Germany) with SupplementMix (C-39216, PromoCell, Germany). We treated HUVECs with sTREM-1 of increasing concentration (0, 0.1, 1.0, 10 μg/mL) for 0.5 hour followed by stimulating the cells by rhTNF-α of 10 ng/mL, or oxLDL of 50 μg/mL for additional 24 hours. Then cells were harvested for Western blotting.

### 2.6 Western blot

For whole cell extraction, cells were lysed using the ProteoJET Mammalian Cell Lysis Reagent (Fermentas, MD). Protein concentration was determined using the BCA method. Equal amounts of protein were subjected to SDS-PAGE and transferred to polyvinylidene fluoride membrane. The membrane was then blocked with 5% nonfat milk in TBST buffer (Tris-buffered saline with 0.2% Tween-20) for 1 hour. The membranes were incubated overnight at 4 °C with primary antibodies, washed 3 times, followed by incubation with secondary antibodies for 1 hour at room temperature. The membranes were rinsed, and the signal was detected using ECL detection system (Millipore, MA).

### 2.7 Statistical analysis

Continuous variables were presented as mean and standard deviation, discrete data were described as median with 25% to 75% range, and categorical data were summarized as frequency with percentage. For categorical clinical variables, we evaluated differences between groups with the Chi-square test followed by Bonferroni correction to account for multiple comparisons. For continuous variables, we evaluated the existence of a normal distribution with the Kolmolgorov–Smirnov test, and applied logarithmic or square-root transformations on continuous variables showing a nonnormal distribution. We analyzed differences among patient groups by one-way analysis of variance (ANOVA) or the Kruskal–Wallis analysis, followed by post-hoc analysis. We determined correlation between variables by the Pearson or Spearman correlation tests, as appropriate. In multivariable stepwise logistic regression analysis, conventional risk factors and sTREM-1 were adjusted for the assessment of CAD determinants. For cell experiments, data represent the average of 6 experiments. A 2-tailed *P* < 0.05 was considered statistically significant. Statistical analysis was performed with SPSS 16.0 for Windows (SPSS, Inc., Chicago, IL).

## 3 Results

### 3.1 Clinical characteristics

Baseline clinical characteristics and biochemical measurements are presented in Table 1. Significant differences with respect to gender and age existed between patients with CAD and those without CAD. The prevalence of cigarette smoking, hypertension, and dyslipidemia was higher in CAD than in control group. The CAD patients had higher levels of systolic pressure, fasting glucose, HbA1c, and lipoprotein (a) but lower levels of high-density lipoprotein cholesterol and apoprotein A as compared with controls. Renal function measurements and uric acid were not obviously different between the 2 groups. Of note, serum high sensitivity C reactive protein (hsCRP) levels were significantly higher in patients with CAD than in controls (5.25 ± 5.53  vs 3.21 ± 4.02 mg/L, *P* < 0.001) (Table 1).

**Table 1:**
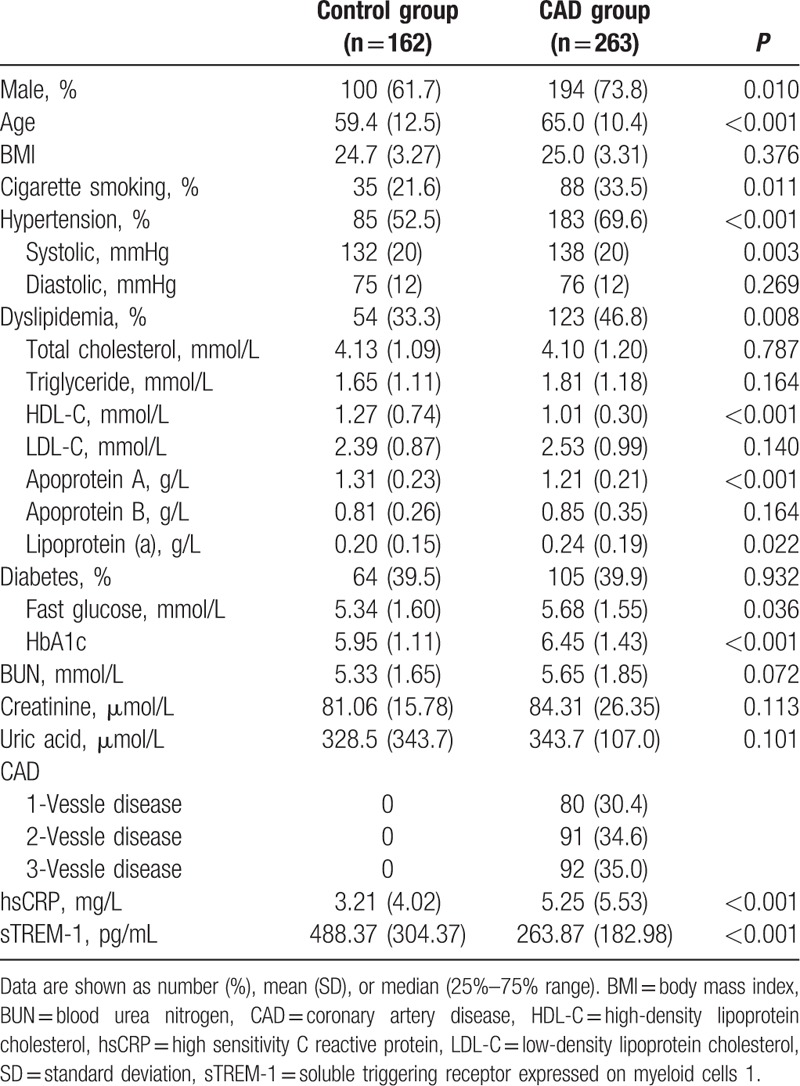
Baseline clinical characteristics and biochemical measurements.

### 3.2 Decreased serum Level of sTREM-1 is associated with the severity of CAD

**Figure 1:**
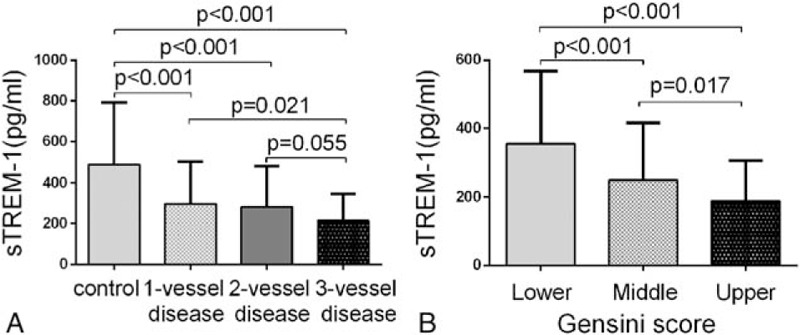
Serum sTREM-1 according to the numbers of diseased coronary artery and tertile of Gensini score. Results are presented as mean ± SD. one-way ANOVA was performed, and LSD analysis was used for multiple comparisons. (A) According to angiography, patients were divided into 4 groups: 0-vessel disease (n = 162), 1-vessel disease (n = 80), 2-vessel disease (n = 91), and 3-vessel disease (n = 92). Serum sTREM-1 concentration negatively correlates to the numbers of diseased artery. (B) Based on their Gensini scores, patients with CAD (CAD group) were trisected into 3 groups: the lower tertile (n = 87, median of Gensini score = 12), the middle tertile (n = 88, median of Gensini score = 35), and the upper tertile (n = 88, median of Gensini score = 82). The serum level of sTREM-1 was significantly decreased in the upper tertile. ANOVA = one-way analysis of variance, CAD = coronary artery disease, LSD = least significant difference, SD = standard deviation, sTREM-1 = soluble triggering receptor expressed on myeloid cells 1.

Importantly, serum sTREM-1 levels were remarkably lower in patients with CAD than in controls (263.87 ± 182.98 vs 488.37 ± 304.37 pg/mL, *P* < 0.001) (Table 1). The patients with CAD were further classified according to the number of diseased coronary arteries and Gensini score. Serum sTREM-1 level was inversely related to the number of diseased coronary arteries (Spearman *r* = −0.413, *P* < 0.001; ANOVA, *P* < 0.001) (Fig. 1A) and severity of stenosis determined by Gensini score (Pearson *r* = −0.336, *P* < 0.001; ANOVA, *P* < 0.001) (Fig. 1B).

### 3.3 Multivariable logistic regression analyses to evaluate the determinants of CAD

Multivariable stepwise logistic regression analysis (adjusting for traditional cardiovascular risk factors, hsCRP, and sTREM-1) revealed that elder age, male, cigarette smoking, dyslipidemia, hsCRP, and sTREM-1 were independent determinants of CAD (Table 2).

**Table 2:**
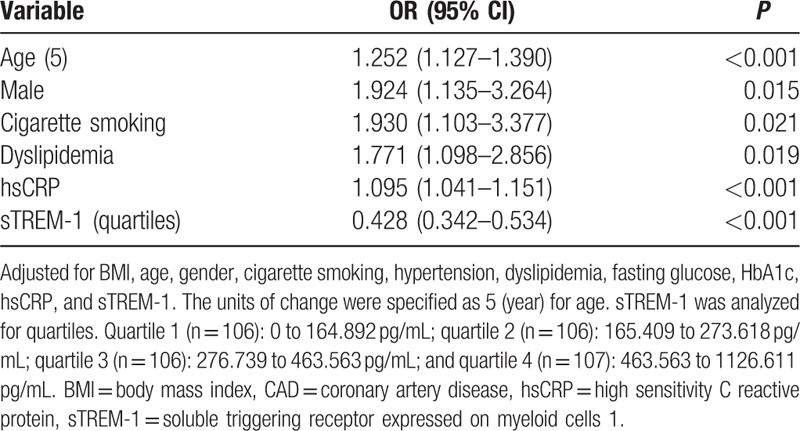
Multivariable stepwise logistic regression analyses of CAD determinants.

Division of all individual into quartiles according to sTREM-1 level provided additional evidence of an association between serum sTREM-1 level and CAD. In the analysis adjusted for traditional cardiovascular risk factors (age, gender, cigarette smoking, hypertension, dyslipidemia, fasting glucose, HbA1c, and hsCRP), upper quartiles of serum sTREM-1 level were associated with an decreased risk of CAD as compared with quartile 1 (OR = 0.448, *P* = 0.030; OR = 0.225, *P* < 0.001 and OR = 0.076, *P* < 0.001, respectively. *P* value for the trend < 0.001) (Table 3).

**Table 3:**
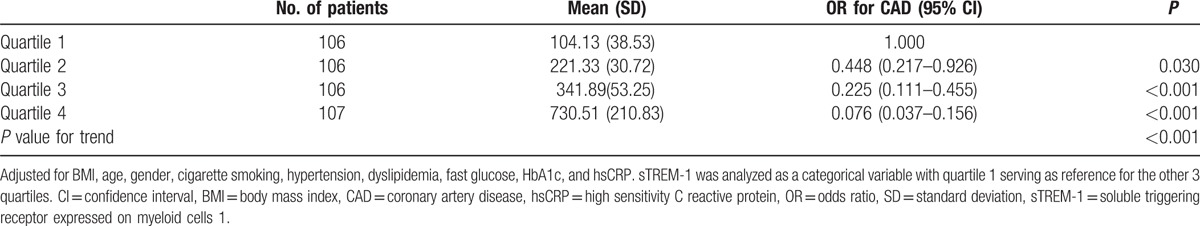
CAD status according to quartiles of sTREM-1.

### 3.4 Serum levels of sTREM-1 is lower in diabetic patients than in nondiabetic controls

The potential relationship between serum sTREM-1 level and traditional risk factor for CAD was explored, such as hypertension, hyperlipidemia, gender, and elder age. In non-CAD control subjects, there was no significant difference in sTREM-1 level between those with hypertension or dyslipidemia and those without. Neither was there any difference in this protein level between male and female, between patients with BMI < 25 kg/m^2^ and those with BMI ≥ 25 kg/m^2^ or between older (age>65 years) and younger participants (data not shown).

Given the crucial association between type 2 diabetes and CAD, we further divided the participants into 4 groups according to the presence or absence of CAD and type 2 diabetes to evaluate the relation of sTREM-1 level and diabetes. Notably, a significant lower serum sTREM-1 level was observed in controls with diabetes than in controls with no diabetes in non-CAD subjects (*P* = 0.048). Patients with both diabetes and CAD had lower serum sTREM-1 level as compared with those with CAD but no diabetes, but the difference did not reach significant level (*P* = 0.314) (Supplementary Table 1). Multivariable stepwise logistic regression analysis revealed that sTREM-1 remained to be an independent determinant of CAD in nondiabetic subjects as well as in diabetic patients (Supplementary Table 2).

### 3.5 sTREM-1 attenuates TNF-α- and oxLDL-induced inflammatory reaction in HUVECs

**Figure 2:**
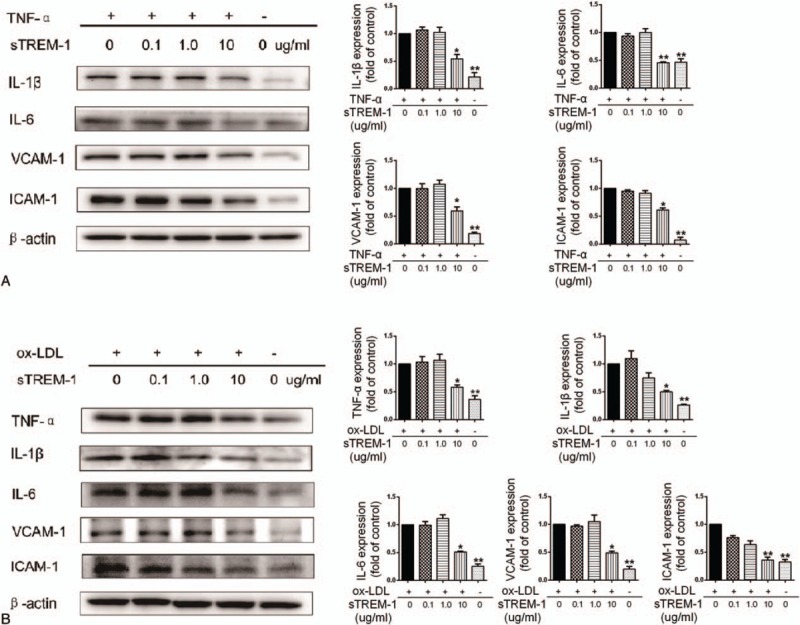
sTREM-1 protects HUVEC from TNF-α and oxLDL in vitro. (A) HUVECs were pretreated with sTREM-1 at 0.1, 1.0, 10 μg/mL or not for 0.5 hour, TNF-α (10 ng/mL) was then added. Cells were cultured for another 24 hours and harvested for Western blotting. Data are means ± SD of 6 independent experiments. ∗*P* < 0.05, ∗∗*P* < 0.01, TNF-α +/sTREM-1 = 0 versus all other values. (B) HUVECs were pretreated with sTREM-1 at 0.1, 1.0, 10 μg/mL or not for 0.5 hour, oxLDL (50 μg/mL) was then added. Cells were cultured for another 24 hours and harvested for Western blotting. Data are means ± SD of 6 independent experiments. ∗*P* < 0.05, ∗∗*P* < 0.01, oxLDL +/sTREM-1 = 0 versus all other values. HUVEC = human umbilical vein endothelial cell, oxLDL = oxidized low-density lipoprotein, TNF-α = tumor necrosis factor-α, sTREM-1 = soluble triggering receptor expressed on myeloid cells 1.

To investigate the biological effect of sTREM-1, we treated HUVECs with TNF-α and oxLDL, with the presence or absence of recombinant sTREM-1 protein of increasing concentrations. After 24 hours, Western blot revealed that TNF-α and oxLDL upregulated the expression of cytokines (IL-1β, IL-6, and TNF-α) and adhesion molecules (VCAM-1 and ICAM-1) as expectation. Notably, sTREM-1 protein concentration-dependently inhibited the inflammatory effects of TNF-α and oxLDL (Fig. 2).

## 4 Discussion

In the present study, we have demonstrated the association of a reduced serum sTREM-1 level with the presence and severity of CAD. In cell experiment, sTREM-1 protein significantly inhibits TNF-α- and oxLDL-induced inflammatory reactions in HUVECs, suggesting that sTREM-1 might be a protective factor against atherogenesis.

Our results appear to be inconsistent with those results reported by Gibot et al^[17]^ and Tao et al,^[20]^ as they revealed that sTREM-1 increased dramatically in sepsis, septic shock, and pneumonia. However, we believe, the acute pathophysiological situation studied above is obviously different from CAD, which is characterized by a chronic vascular inflammatory disorder.^[1,3]^ Despite the increase of serum sTREM-1 level in acute phase, these authors also showed that sTREM-1 levels were significantly lower at admission in nonsurvivors than in survivors, suggesting the association of decreased serum sTREM-1 level with poor prognosis in severe inflammatory pathophysiology.^[18,20,26]^ Giamarellos-Bourboulis et al^[27]^ exhibited that alteration of serum sTREM-1 level was in agreement to the change of IL-10 level in a cohort of 90 patients suffering from a ventilator-associated pneumonia, and a good correlation was observed between the ratios IL-10/TNF-α and sTREM-1/TNF-α, IL-10/IL-6 and sTREM-1/IL-6, and IL-10/IL-8 and sTREM-1/IL-8, suggesting a potential antiinflammatory role of sTREM-1. However, Hermus et al^[28]^ have also reported an elevated serum sTREM-1 level in patients with CAD versus those with no CAD. The reason regarding the inconsistency between their study and ours is unclear.

The role of sTREM-1 as an antiinflammatory protein is further supported by experimental studies. LP17, a synthetic peptide mimicking highly conserved extracellular domain of TREM-1, decreased the cytokine production in human monocytes and protected septic animals from hyper-responsiveness and death.^[11,29]^ A chimeric protein TREM-1/IgG1 (equivalent to the extracellular domain of TREM-1) blocked TREM-1 activation during endotoxemia and peritonitis in C57BL/6 mice.^[13]^

In our study, we have shown that sTREM-1 can inhibit the expression of IL-1β, IL-6, TNF-α, VCAM-1, and ICAM-1 in HUVECs activated by TNF-α or oxLDL, indicating a protective role of sTREM-1 in the inflammatory process of endothelium. Thus, it is hypothesized that sTREM-1 may act as a decoy receptor to neutralize ligands of TREM-1 receptor during inflammatory reactions and downregulate the TREM-1/TREM-1 activates cells through DNAX-activating protein of 12 kDa activation pathway.^[12]^ Such protective effect of sTREM-1 is somehow crippled due to reduced levels in vascular atherosclerosis. Wu et al^[30]^ reported that high mobility group box (HMGB) 1 maybe a TREM-1 ligand in Kupffer cells, so we tried to determine whether HMGB1 was remarkably released by TNF-α or oxLDL stimulated HUVECs, and found that HMGB1 level was significantly higher in the culture supernatants of HUVECs triggered by TNF-α and oxLDL (Supplementary Fig. 1). Although the exact reaction between sTREM-1 and HMGB1 is not clarified yet and is worthy a deeper look.

In conclusion, we have shown the association of reduced serum sTREM-1 level with the presence and severity of CAD, and an antiinflammatory function of sTREM-1 protein in endothelial cells.

Finally, we do recognize some limitations in the present study. First, this study is actually a cross-sectional one, which does not have the power for prediction. Further large scale, randomized study is needed to confirm our results. Second, we observed an association between serum sTREM-1 level and diabetes, while the relationship was not explored by experiments in current study. Third, we demonstrated the effect of sTREM-1 in endothelial cells but not vascular smooth muscle cells. As the progress of atherosclerosis is complicated which involves the interaction of both endothelium cells and underlying smooth muscle cells, further researches should be done to probe the relationship among sTREM-1, endothelial cells, and smooth muscle cells.

## Acknowledgments

The authors thank the National Natural Science Foundation of China 81070178 and 81370256, and the Shanghai Science and Technology Key Project grant 12JC1406300 (to RT) for the support.

## Supplementary Material

Supplemental Digital Content
