# Supplementary material for: Association of decreased serum sTREM-1 level with the severity of coronary artery disease: Inhibitory effect of sTREM-1 on TNF-α- and oxLDL-induced inflammatory reactions in endothelial cells
Source: Medicine (Baltimore). 2016 Sep 16;95(37):e4693. doi: 10.1097/MD.0000000000004693 (PMC5402559; doi:10.1097/MD.0000000000004693)
Supplement: Supplemental Digital Content [file medi-95-e4693-s001.doc]

**Supplementary Figure 1.** **HMGB1 is released by HUVECs activated by TNF-α and oxLDL.** HUVECs were treated with hrTNF-α (10░ng/ml) or oxLDL (50░ug/ml) for 24░h and the culture supernatants were harvested for Western Blotting analysis of HMGB1.


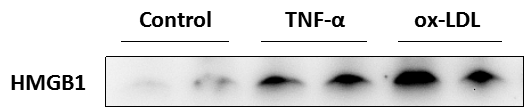


**Supplementary Table 1. Baseline clinical characteristics and biochemical measurements of subgroup patients**

|  | Nondiabetic patients | | |  | Diabetic patients | | |
| --- | --- | --- | --- | --- | --- | --- | --- |
|  | No CAD  Group I (n=98) | CAD  Group II (n=158) | P value |  | No CAD  Group III (n=64) | CAD  Group IV (n=105) | P value |
| Men (%) | 59 (60.2) | 120 (75.9) | 0.011 |  | 41 (64.1) | 74 (70.5) | 0.400 |
| Age (years) | 58.8 (13.3) | 65.1 (9.98) | <0.001 |  | 60.3 (11.2) | 65.0 (11.0) | 0.009 |
| BMI | 24.8 (3.3) | 24.7 (3.3) | 0.859 |  | 24.6 (3.2) | 25.4 (3.3) | 0.103 |
| Cigarette smoking (%) | 23 (23.5) | 52 (32.9) | 0.121 |  | 12 (18.8) | 36 (34.3) | 0.035 |
| Hypertension (%) | 39 (39.8) | 108 (68.4) | <0.001 |  | 46 (71.9) | 75 (71.4) | 0.950 |
| Blood pressure |  |  |  |  |  |  |  |
| Systolic (mmHg) | 133 (18) | 137 (20) | 0.137 |  | 130 (22) | 139 (20) | 0.005 |
| Diastolic (mmHg) | 76 (12) | 76 (12) | 0.696 |  | 74 (11) | 76 (12) | 0.195 |
| Dislipidemia (%) | 20 (20.4) | 75 (47.5) | <0.001 |  | 34 (53.1) | 48 (45.7) | 0.428 |
| Total cholesterol (mmol/l) | 4.15 (1.00) | 4.14 (1.21) | 0.908 |  | 4.10 (1.22) | 4.05 (1.18 ) | 0.784 |
| Triglyceride (mmol/l) | 1.37 (0.56) | 1.66 (0.86) | 0.001 |  | 2.07 (1.54) | 2.03 (1.51) | 0.861^a^ |
| HDL-C (mmol/l) | 1.27 (0.30) | 1.04 (0.35) | <0.001 |  | 1.27 (1.13) | 0.96 (0.22) | 0.034^a^ |
| LDL-C (mmol/l) | 2.38 (0.92) | 2.58 (1.01) | 0.117 |  | 2.40 (0.79) | 2.46 (0.96) | 0.716 |
| Apo(A) (g/l) | 1.35 (0.23) | 1.22 (0.19) | <0.001 |  | 1.25 (0.22) | 1.19 (0.23) | 0.121 |
| Apo(B) (g/l) | 0.78 (0.23) | 0.86 (0.40) | 0.087 |  | 0.84 (0.30) | 0.84 (0.26) | 0.917 |
| lipoprotein（a) (g/l) | 0.19 (0.15) | 0.24 (0.20) | 0.026 |  | 0.21 (0.14) | 0.23 (0.19) | 0.408 |
| Fast Glucose (mmol/l) | 4.85 (0.63) | 4.86 (0.60) | 0.910 |  | 6.10 (2.22) | 6.90 (1.73) | 0.009^b^ |
| HbA1c (%) | 5.52 (0.69) | 5.77 (0.95) | 0.026 |  | 6.61 (1.30) | 7.48 (1.41) | <0.001^b^ |
| BUN (mmol/l) | 5.24 (1.86) | 5.51 (1.74) | 0.248 |  | 5.45 (1.28) | 5.85 (2.00) | 0.154 |
| Cr (umol/l) | 80.64 (14.82) | 85.37 (26.31) | 0.105 |  | 81.70 (17.25) | 82.7 (26.4) | 0.786 |
| UA (umol/l) | 326.88 (85.92) | 353.0 (109.8) | 0.046 |  | 330.95 (76.78) | 329.6 (101.5) | 0.925 |
| CAD |  |  |  |  |  |  |  |
| 1-vessle disease | 0 | 59 (37.3) |  |  | 0 | 21 (20.0) |  |
| 2-vessle disease | 0 | 51 (32.3) |  |  | 0 | 40 (38.1) |  |
| 3-vessle disease | 0 | 48 (30.3) |  |  | 0 | 44 (41.9) |  |
| HsCRP(mg/l) | 2.38(3.45) | 4.06(4.81) | 0.001 |  | 4.47(4.50) | 7.03(6.07) | 0.002^b^ |
| sTREM-1 (pg/ml) | 526.51 (302.28) | 275.78 (191.93) | <0.001 |  | 429.98(300.56) | 245.93 (167.91) | <0.001 |

Data are shown as number (%), mean (SD) or median (25%~75% range).

Symbols: ^a^p<0.05, ^b^p<0.001 (patients with CAD and diabetes vs. patients with CAD but no diabetes).

**Supplementary Table 2．Multivariable stepwise logistic regression analyses of CAD determinants in non-diabetic and diabetic subjects.**

| Variable | OR (95% CI) | P value |
| --- | --- | --- |
| Nondiabetic patients: |  |  |
| Male | 2.116 (1.006~4.450) | 0.048 |
| Hypertension | 2.787 (1.447~5.368) | 0.002 |
| Dyslipidemia | 2.547(1.257~5.161) | 0.009 |
| HDL-C | 0.207(0.065~0.657) | 0.008 |
| hsCRP | 1.097 (1.010~1.192) | 0.028 |
| sTREM-1 (quartiles) | 0.367 (0.264~0.508) | <0.001 |
| Diabetic patients: |  |  |
| Age (5) | 1.309 (1.099~1.561) | 0.003 |
| Cigarette smoking | 3.650(1.429~9.321) | 0.007 |
| HbA1c | 1.713 (1.249~2.349) | 0.001 |
| hsCRP | 1.116(1.036~1.202) | 0.004 |
| sTREM-1 (quartiles) | 0.520 (0.361~0.750) | <0.001 |

Adjusted for age, gender, cigarette smoking, hypertension, dyslipidemia, fast glucose, HbA1c, TC, TG, HDL-C, LDL-C, hsCRP and sTREM-1. The units of change were specified as 5 (year) for age. sTREM-1 was analysed for quartiles.
